# RETRACTION: PLK2 Phosphorylates and Inhibits Enriched TAp73 in Human Osteosarcoma Cells

**DOI:** 10.1002/cam4.72044

**Published:** 2026-06-12

## Abstract

**RETRACTION**: HuZ. B. , LiaoX. H. , XuZ. Y. , YangX. , DongC. , JinA. M. and LuH. , “PLK2 Phosphorylates and Inhibits Enriched TAp73 in Human Osteosarcoma Cells,” Cancer Medicine 5, no. 1 (2016): 74‐87, 10.1002/cam4.558.26625870 PMC4708894

The above article, published online on 02 December 2015 in Wiley Online Library (wileyonlinelibrary.com), has been retracted by agreement between the journal Editor‐in‐Chief, Stephen Tait; and John Wiley and Sons Ltd. The retraction has been agreed upon following concerns raised by a third party. An investigation identified the duplication of bands within Figure 3F. Further duplications were observed between panels presented in Figure 8. An additional duplication was observed between the IB TAp73, 15 *μ*g/mL Cis bands shown in Figure 2A and bands presented in another article published elsewhere by some of the same authors. In this case, the duplicate bands are shown to represent different proteins. The authors were contacted for their comments and supporting data but did not respond. The editors consider the results and conclusions to be compromised. The authors did not respond to our notice of retraction.

**RETRACTION**: Z. B. Hu , X. H. Liao , Z. Y. Xu , X. Yang , C. Dong , A. M. Jin and H. Lu , “PLK2 Phosphorylates and Inhibits Enriched TAp73 in Human Osteosarcoma Cells,” Cancer Medicine 5, no. 1 (2016): 74‐87, 10.1002/cam4.558.26625870 PMC4708894

The above article, published online on 02 December 2015 in Wiley Online Library (wileyonlinelibrary.com), has been retracted by agreement between the journal Editor‐in‐Chief, Stephen Tait; and John Wiley and Sons Ltd. The retraction has been agreed upon following concerns raised by a third party. An investigation identified the duplication of bands within Figure 3F. Further duplications were observed between panels presented in Figure 8. An additional duplication was observed between the IB TAp73, 15 *μ*g/mL Cis bands shown in Figure 2A and bands presented in another article published elsewhere by some of the same authors. In this case, the duplicate bands are shown to represent different proteins. The authors were contacted for their comments and supporting data but did not respond. The editors consider the results and conclusions to be compromised. The authors did not respond to our notice of retraction.

